# Surgical peritoneal stress creates a pro-metastatic niche promoting resistance to apoptosis via IL-8

**DOI:** 10.1186/s12967-018-1643-z

**Published:** 2018-10-03

**Authors:** Jennifer Pasquier, Fabien Vidal, Jessica Hoarau-Véchot, Claire Bonneau, Emile Daraï, Cyril Touboul, Arash Rafii

**Affiliations:** 1 Stem Cell and Microenvironment Laboratory, Weill Cornell Medical College in Qatar, Education City, Qatar Foundation, PO: 24144, Doha, Qatar; 2 Department Genetic Medicine, Weill Cornell Medical College, New York, NY USA; 3 INSERM U955, Equipe 7, Créteil, France; 4 Service de Gynécologie Obstétrique, Hopital Tenon (Assistance Publique–Hôpitaux de Paris), 4 rue de la Chine, 75020 Paris, France; 5 Service de Gynécologie-Obstétrique et Médecine de la Reproduction, Faculté de médecine de Créteil UPEC–Paris XII, Centre Hospitalier Intercommunal de Créteil, 40 Avenue de Verdun, 94000 Créteil, France; 6 Service de chirurgie Gynécologique, Hôpital Foch, 92100 Suresnes, France

**Keywords:** Ovarian cancer, Chemoresistance, Surgery, Tumor microenvironment, IL8

## Abstract

**Background:**

The mainstay of treatment of advanced ovarian cancer (AOC) involves chemotherapy, and debulking surgery. However, despite optimal surgical procedure and adjuvant chemotherapy, 60% of patients with AOC will relapse within 5 years. Most recurrences occur in the peritoneal cavity, suggesting the existence of occult sanctuaries where ovarian cancer cells (OCC) are protected. In murine models, surgical stress favors tumor growth; however, it has never been established that surgery may affect OCC sensitivity to subsequent chemotherapy. In this study, we investigated how the surgical stress could affect the chemosensitivity of OCC.

**Methods:**

To avoid bias due to tumor burden in peritoneal cavity and duration of surgery, we used peritoneal biopsies from patients without a malignancy at precise time points. During laparotomies, peritoneal biopsies at the incision site were performed at the time of incision (H0 sample) and 1 h after initiation of surgery (H1 sample). We evaluated the chemoresistance to Taxol (0–20 µM) induced by H0 or H1 incubation (24 h) in two ovarian cancer cell lines OVCAR3 and SKOV3 and a primary cancer cell lines derived in our laboratory.

**Results:**

Our results indicate that stressed peritoneum overexpressed cytokines, resulting in OCC increased resistance to therapy. Among these cytokines, IL8 was responsible for the resistance to apoptosis through the AKT pathway activation. Chemoresistance in OCC persists through the establishment of an autocrine IL8 loop. Finally, in a cohort of 32 patients, we showed an impact of IL8 tumoral overexpression on chemosensitivity and survival outcomes with a significant association to earlier recurrence.

**Conclusions:**

Our study demonstrated that precision surgery where targeted treatment would be used in combination with surgery is essential to obtain better tumor control.

**Electronic supplementary material:**

The online version of this article (10.1186/s12967-018-1643-z) contains supplementary material, which is available to authorized users.

## Background

Ovarian cancer is the deadliest gynecologic malignancy due to early extensive spread to the peritoneal cavity. Despite optimal surgeries and initial chemosensitivity leading in most patients to complete cytoreduction (CC-0), peritoneal recurrences will be the main site of recurrence impacting patient’ survival [1]. In referee teams, while CC-0 is achieved in up to 80% of cases [2], 60% of patients with AOC will relapse within 5 years of initial diagnosis [3]. The location of recurrences within the peritoneal cavity strongly suggests the existence of occult sanctuaries where cancer cells are protected against therapy.

We acknowledge that complete cytoreduction is theoretical in most patients, particularly in case of extended peritoneal carcinomatosis and ascites and the surgical paradigm remains macroscopic. Hence, despite macroscopic CC-0 surgery [4], a variable amount of microscopic residual disease will be left in place. Several studies have suggested that the crosstalk between cancer cells and host’s stromal cells may participate in the establishment of a permissive tumoral environment that subsequently favors residual disease growth and chemoresistance [5–11]. However, only few studies have provided a global approach, considering the macro-environmental changes associated with surgery [12–14]. Beyond cell–cell interactions, peritoneal trauma induced by surgery may also participate in the constitution of a niche for residual cancer cells through overproduction of secreted factors. In murine models of ovarian cancer surgical stress favors tumor growth through induction of angiogenesis and OCCs proliferation and adhesion [15, 16] through activation of the β adrenergic pathway and expression of VEGF, MMP-2 and various inflammatory cytokines, including IL6 and IL8 [17]. However, it has never been established that surgery may influence OCC sensitivity to subsequent chemotherapy.

In this study, using a tailored model we investigated the role of surgical stress on OCC resistance to taxane-based chemotherapy. We show that stressed peritoneum overexpressed cytokines such as IL8, resulting in OCC increased resistance to therapy through the activation of AKT pathway.

## Methods

### Cell cultures

Ovarian cancer cells lines SKOV3, OVCAR3, were purchased from ATCC and cultured following ATCC recommendations (ATCC, Manassas, VA, USA). A primary ovarian cancer cell line was derived in our laboratory from ascites of a patient with Stage III serous adenocarcinoma (APOCC) (REF papier utilize avant). The cell lines were cultured in DMEM high glucose (Hyclone, Thermo Scientific), 10% FBS (Hyclone, Thermo Scientific), 1% Penicillin–Streptomycin-Amphotericyn B solution (Sigma), 1× Non-Essential Amino-Acid (Hyclone, Thermo Scientific) and 1% l-glutamine. Cultures were incubated in humidified 5% CO_2_ incubators at 37 °C and the media was replaced every 3 days.

### H0 and H1 sampling

Normal peritoneal samples of patients with suspicious ovarian tumor but finally benign were used in this study. Patients included in the PELVIMASS protocol, which was accepted by the French Research Ethics Committee chair (CPP No. 2016-A01381-42), signed informed consent. All conditions required surgical treatment with laparotomy. In each patient, 4 cm^2^ peritoneal samples were harvested at a required peritoneal incision site (broad ligaments) at the time of incision (H0 specimen) and 1 h after initiation of the procedure (H1 specimen). Peritoneal samples were incubated at 37 °C in DMEM low glucose for 6 h. Media were subsequently filtered, aliquoted and stored at − 80 °C.

### Cell proliferation assay

Cells were plated at 50,000 cells per well in a 6 well plate in medium without FBS. Cells were then counted with a hemocytometer for the following 6 days every 2 days. Two wells were counted per conditions. The experiment was performed in triplicates. All functional assays were performed using conditioned media from three to five different patients.

### Migration assay

Migration was assessed by wound closure assay as previously described [5]. Briefly, Cells cultured at confluence in 24-well plates were scratched with a small tip along the ruler. Cells were then cultured for 6, 24 or 48 h in starvation media with or without MPs. The distances between the edges of the scratch were measured at 0 h and 6, 24 or 48 h after scratching. Data are represented as rate of wound closure.

### Tube formation assay

A Matrigel-based capillary-genesis assay was performed on cells to assess their ability to form an organized tubular network as previously described [18]. Briefly, cells were starved for 6 h prior the experiment. Then 100,000 cells were cultured on 250 μl of Matrigel (BD bioscience). The degree of tube formation was quantified at different time-points by measuring the intersection of tubes in three randomly chosen fields from each well using ImageJ.

### Flow cytometry

Fluorescence (FL) was quantified on a SORP FACSAria2 (BD Biosciences). Data were processed with FACS Diva 6.3 software (BD Biosciences) as previously described [19]. Doublets were excluded by FSC-W × FSC-H and SSC-W × SSC-H analysis; calcein-AM and Annexin V were acquired with 488 nm blue laser and 510/50 nm emission, PI was acquired 488 nm blue laser and 670/14 nm emission. Charts display the median of fluorescence intensity (mfi) relative to control. Single stained channels were used for compensation and fluorophore minus one (FMO) controls were used for gating. 20,000 events were acquired per sample.

### Confocal microscopy

Confocal microscopy was performed on fixed cells in 3.7% formaldehyde. Cells were stained with a 50 µg/ml AF647-conjugated phalloidin (Sigma) to label actin filaments. Slides were mounted in a mounting media SlowFade^®^ Gold Antifade Reagent with DAPI (Invitrogen). Imaging was performed using a Zeiss confocal Laser Scanning Microscope 710 (Carl Zeiss). Post-acquisition image analysis was performed with Zeiss LSM Image Browser Version 4.2.0.121 (Carl Zeiss).

### Calcein-AM staining

Calcein-AM indicates intracellular esterase activity. Cells were washed twice with Phosphate buffer saline (PBS). Cells were next stained with the 2 μM of calcein-green-AM (Molecular Probes, Invitrogen, Leiden NL) for 45 min at 37° 5% CO_2_ according to manufacturers instructions. They were then immediately analyzed by FACS on a SORP FACSAria2 (BD Bioscience, San Jose, CA) as described.

### Western blot analysis

Western blot were carried out as previously described [7]. Immunostaining was carried out using a rabbit monoclonal caspase3, caspase9, actin and PhosphoAKT antibody (1/1000, Cells signaling) and a secondary polyclonal mouse anti-rabbit antibody HRP conjugated (1/2000, cell signalling). Blots were developed using HRP and chemiluminescent peroxidase substrate (#CPS1120, Sigma). Data were collected using Geliance CCD camera (Perkin Elmer), and analyzed using ImageJ software (NIH).

### RT-PCR analysis

Total RNA was extracted from cells cultures using Trizol. After genomic DNA removal by DNase digestion (Turbo DNA free kit, Applied Biosystems), total RNA (1 µg) was reverse transcribed with oligodT (Promega) using the Superscript III First-Strand Synthesis SuperMix (Invitrogen). PCR analysis was performed as previously described [20] with a MasterCycler apparatus (Eppendorf) from 2 µl of cDNA using primers from IDT (Table 1).

**Table 1:** Comparative demographics between chemoresistant and platinium sensitive patients

	Platinium resistant subgroup (n = 16)	Platinium sensitive subgroup (n = 12)	p-value
Age (years)	60.4 (± 12.1)	63.8 (± 6.6)	0.38
BMI	24.1 (± 4.6)	23.8 (± 3.2)	0.82
CA 125 (U/ml) at baseline	2600 (± 3098.2)	1235 (± 1252.0)	0.16
Histological type			
Serous	87.5% (n = 14)	66.7% (n = 8)	0.58
Undifferentiated	6.25% (n = 1)	16.7% (n = 2)	
Mucinous	0	8.3% (n = 1)	
Endometrioid	6.25% (n = 1)	8.3% (n = 1)	
FIGO stage			
IIIC	81.2% (n = 13)	100% (n = 12)	0.11
IV	18.8% (n = 3)	0	
Count of NAC courses	4 (3–6)	3 (3–7)	0.34
Total count of chemotherapy courses	7 (3–12)	6 (3–9)	0.15
Delay between diagnosis and surgical debulking (months)	4.3 (± 1.2)	3.9 (± 1.2)	0.43
Completeness of cytoreduction score^a^			
CC-0	68.7% (n = 11)	83.4% (n = 10)	0.38
CC-1	6.3% (n = 1)	0	
CC-2	18.7% (n = 3)	8.3% (n = 1)	
CC-3	6.3% (n = 1)	8.3% (n = 1)	
Mean IL8 expression on tumor sample	31.3%	11.0%	0.004**
Duration of surgical procedure (min)	428 (± 139)	310 (± 82)	0.07
Overall survival (months)	31.9	78.9	< 10^−4^***

*BMI* body mass index, *NAC* neoadjuvant chemotherapy

^a^A CC-0 score indicates a complete disease removal; a CC-1 score indicates that tumor nodules persisting after cytoreduction were < 2.5 mm in diameter; a CC-2 score indicates residual tumor nodules between 2.5 mm and 25 mm in diameter; a CC-3 score indicates residual tumor nodules > 25 mm in diameter or a confluence of unresectable tumor nodules at any site within the abdomen or pelvis

### Chemoresistance and cell viability study (MTT assay)

Cell viability was examined with an MTT assay [21]. Briefly, 24 h after treatment with doxorubicin, 10% of MTT reagent was added to each well to a final concentration of 500 μg/ml, and the cells were incubated for 4 h at 37 °C. 100 μl of DMSO were added to each well. Optical density was read at 570 nm versus 630 with an EnVision multilabel reader (PerkinElmer, Massachusetts, USA). 3 triplicates were performed per condition.

### Study population

We reviewed tumor samples from 32 patients with advanced ovarian cancer (AOC) referred to Tenon Hospital (Paris, France) from January 2004 to July 2011. This study protocol was approved by the chair of the ethics committee of Paris VI, allowing the use of tumor tissues and medical chart of patients treated for ovarian cancer in our center. They all received platinium and taxane based neoadjuvant chemotherapy, followed by interval debulking surgery. All data, including demographics, FIGO stage, histological type and grade, and treatment modalities were collected retrospectively. Completeness of cytoreduction score was used to evaluate residual disease et the end of debulking surgery [4]. During follow up, patients who relapsed within 12 months following last chemotherapy regimen or suffering from refractory disease were considered chemo resistant.

### Immunohistochemistry protocol for tumor samples

Immunohistochemistry staining were performed as previously described [22]. For each patient, we selected the most relevant tumor paraffin blocks from interval debulking surgery. Paraffin-embedded sections were deparaffinized in xylene and rehydrated in graded alcohol. Immunostaining was perfomed manually, using the Dako Envision + Dual Link System-HRP kit and anti-IL8 primary antibodies (mouse monoclonal to IL8, clone 807, ref. Ab18672, Abcam, UK). Immunostaining specificity was verified with a control antibody and a positive tissue control. All slides were counterstained with hematoxylin. Microscopic analyses were performed using a Nikon Eclipse 90i microscope (Nikon, Nikon Instrument B.V., France). Representative photographs (20× magnification) of tumor immunostaining were performed using the NIS-Element BR software package (Nikon, Nikon Instrument B.V., France). Standardized quantitative analysis of IL8 immunostaining was based on Image J software (National Institutes of health, USA). Briefly, a grid delimitating 10,000 μm^2^ squares was applied to each photograph, and 3 randomly selected squares were analyzed. We used the Image J cell counter plugin to count the stained an unstained tumor cells within each square. IL8 expression was defined as the rate of stained tumor cells.

### Statistical analysis

All quantitative data were expressed as mean ± standard error of the mean (SEM). Statistical analysis was performed using SigmaPlot 11 (Systat Software Inc., Chicago, IL). A Shapiro–Wilk normality test, with a p = 0.05 rejection value, was used to test normal distribution of data prior further analysis. All pairwise multiple comparisons were performed by one way ANOVA followed by Holm–Sidak posthoc tests for data with normal distribution or by Kruskal–Wallis analysis of variance on ranks followed by Tukey posthoc tests, in case of failed normality test. Paired comparisons were performed by Student’s t-tests or by Mann–Whitney rank sum tests in case of unequal variance or failed normality test. Clinical data were anonymized and de-identified prior to analysis. Overall survival (OS) was computed from the date of initial diagnosis. Disease free survival (DFS) was computed from the completion of first line treatment. The first-event corresponded to death of any cause for OS and to relapse or death for DFS. OS and DFS curves were achieved using Kaplan–Meier analysis. The Cox proportional hazard regression model was used for multivariate analysis. All variables associated with *p *<* 0.10* on univariate analysis were included in the model. Statistical significance was accepted for p < 0.05 (*), p < 0.01 (**) or p < 0.001 (***). All experiments were performed in triplicates.

## Results

### Surgical peritoneal stress induces chemoresistance and pro-metastatic phenotype in ovarian cancer cells

**Fig. 1:**
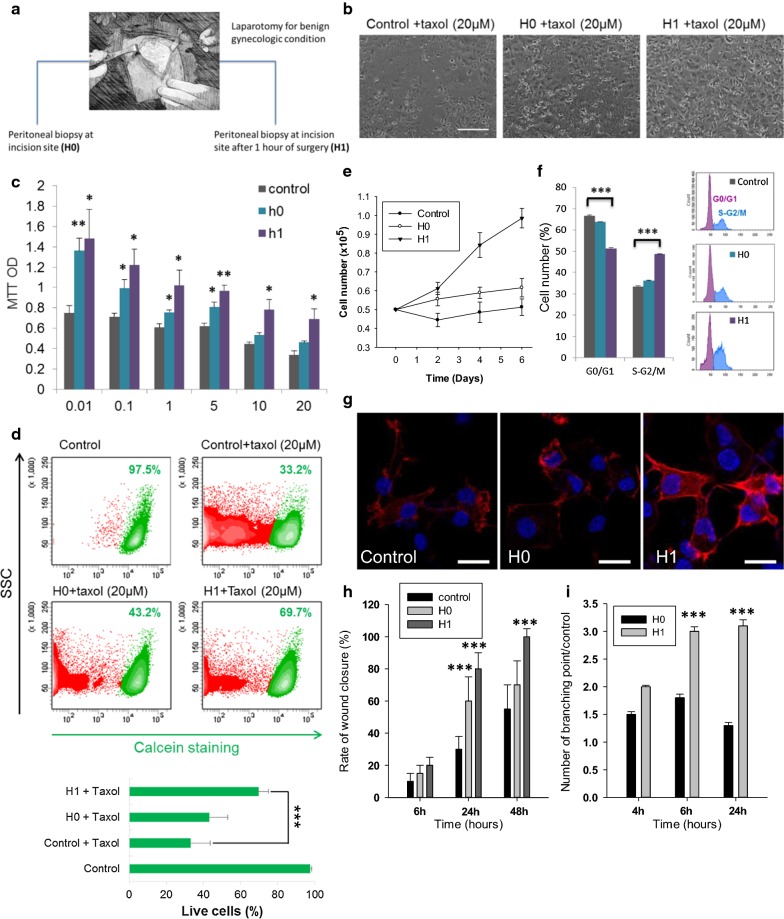
**a** Schematic representation of the procedure. During a laparotomy performed for benign gynecologic condition, a peritoneal biopsy is done at the incision site (H0) and a second one is done 1 h after the incision (H1). **b** Phase contrast microscopy imaging. Ovarian cancer cells (APOCC) treated with Taxol (0–20 µM) for 24 h in presence of H0 (middle picture), H1 (right picture) or nothing (left picture). Scale bar: 500 µm. **c** MTT assay. APOCC were treated with Taxol (0.01–20 µM) in presence of H0 (green), H1 (purple) or nothing (grey). After 48 h a MTT assay was performed. The histogram represents the mean OD MTT. **d** Cell viability. APOCC untreated (control, top left), treated with Taxol (20 µM) alone (top right) or treated with Taxol (20 µM) in presence of H0 (bottom left) or H1 (bottom right) were stained with calcein-AM. Calcein fluorescence was acquired by flow cytometry. **e** Proliferation assay. APOCC were plated and counted every 2 days in presence or not of H0 or H1 during 6 days. **f** Cell cycle analysis. APOCC were treated with H0 or H1 for 48 h and position in cell cycle were evaluated with NIM-DAPI by flow cytometry. The percentage of cells in phase G0/G1 (purple) and in G2/M (blue) is represented on the histogram. **g** F-actin polymerisation in APOCC. APOCC were grown on glass bottom slides with H0 or H1 and actin cytosqueletton was revealed by a phalloïdin-fluorescein (1 μg/ml) labelling (red). Scale bar 15 μm. **h** Wound closure assay. Migration ability of APOCC was tested after a scratch in presence of different H0 or H1. The histogram represents the percentage of wound closure after 6, 24 or 48 h. **i** APOCC plasticity on Matrigel. APOCC were seeded on a 96-wells plate, coated with Matrigel in presence or absence of H0 or H1. Microscopic pictures of cellular networks after H0 and H1 stimulation were taken after 4, 6 and 24 h of culture. Quantitative evaluation of the cellular interconnection are presented on the histogram. The evaluation was made by counting on 10 different fields. The results are expressed as means treated/mean control with standard error. Experiments were performed in triplicate. *p < 0.05, **p < 0.01 or ***p < 0.001

We modeled peritoneal stress using peritoneal biopsies from patients undergoing staging procedures for suspicious early stage ovarian cancer with no extension to the peritoneal cavity (we kept only normal peritoneal biopsies of benign patients). During laparotomies, peritoneal biopsies were performed at the time of incision (H0 sample) and 1 h after initiation of surgery (H1 sample; Fig. 1a). A pathology examination confirmed the absence of tumoral cells within the peritoneal biopsies. The biopsies were then incubated for 6 h at 37 °C in DMEM low glucose and the obtained conditioned media used for the experimental procedures. We evaluated the effect of the peritoneal conditioned media at H0 and H1 on a primary ovarian cancer cell line derived in our laboratory from ascites of a patient with FIGO stage IIIC serous adenocarcinoma (APOCC) and two commercially available ovarian cancer cell lines (OVCAR3 and SKOV3).

To evaluate the effect of peritoneal conditioned media, we treated previously incubated ovarian cancer cells (OCC) with Taxol (0–20 µM). Both H0 and H1 induced resistance to Taxol compared to regular medium (APOCC, Fig. 1b–d and SKOV3 and OVCAR3, Additional file 1: Figure S1A, B). At high concentrations of Taxol (10 and 20 µM) H1 media was significantly more efficient than H0 to promote OCC survival (Fig. 1c, d). H1 media improved the proliferation of APOCC, OVCAR3 and SKOV3 cultured in starving condition (Fig. 1e and Additional file 1: Figure S1C). APOCC cell cycle analysis demonstrated an increase in S phase and G2/M when cultured with H1 (33.47% ± 0.35, 35.13% ± 0.23 and 48.66% ± 0.21 for control, H0 and H1 respectively p < 0.001; Fig. 1f).

To evaluate the impact on cancer cell phenotype, we performed confocal microscopy imaging of APOCC treated with H0 or H1. We observed an increase in F-actin stress fibers in the periphery of the cells (Fig. 1g). The stress fibers and filopods formation required for cancer cells invasion into tissues were observed only during H1 treatment. Accordingly, H1 media induced increased migration compared to H0 for all cell lines (Fig. 1h). We then evaluated cellular plasticity under H0 and H1 treatment by quantifying tube formation on matrigel after 24 h of culture (Fig. 1i). Tube formation was observed as early as 4 h after treatment with H0 or H1; however, (i) the number of tubes and the kinetic of tube formation were lower with H0 and (ii) the persistence of tubes at 6 and 24 h was only observed after H1 treatments. Overall, OCC incubation with peritoneal conditioned media resulted in a pro-metastatic phenotype and increased resistance to taxane therapy.

### Effect of surgical peritoneal stress on cytokines secretion

**Fig. 2:**
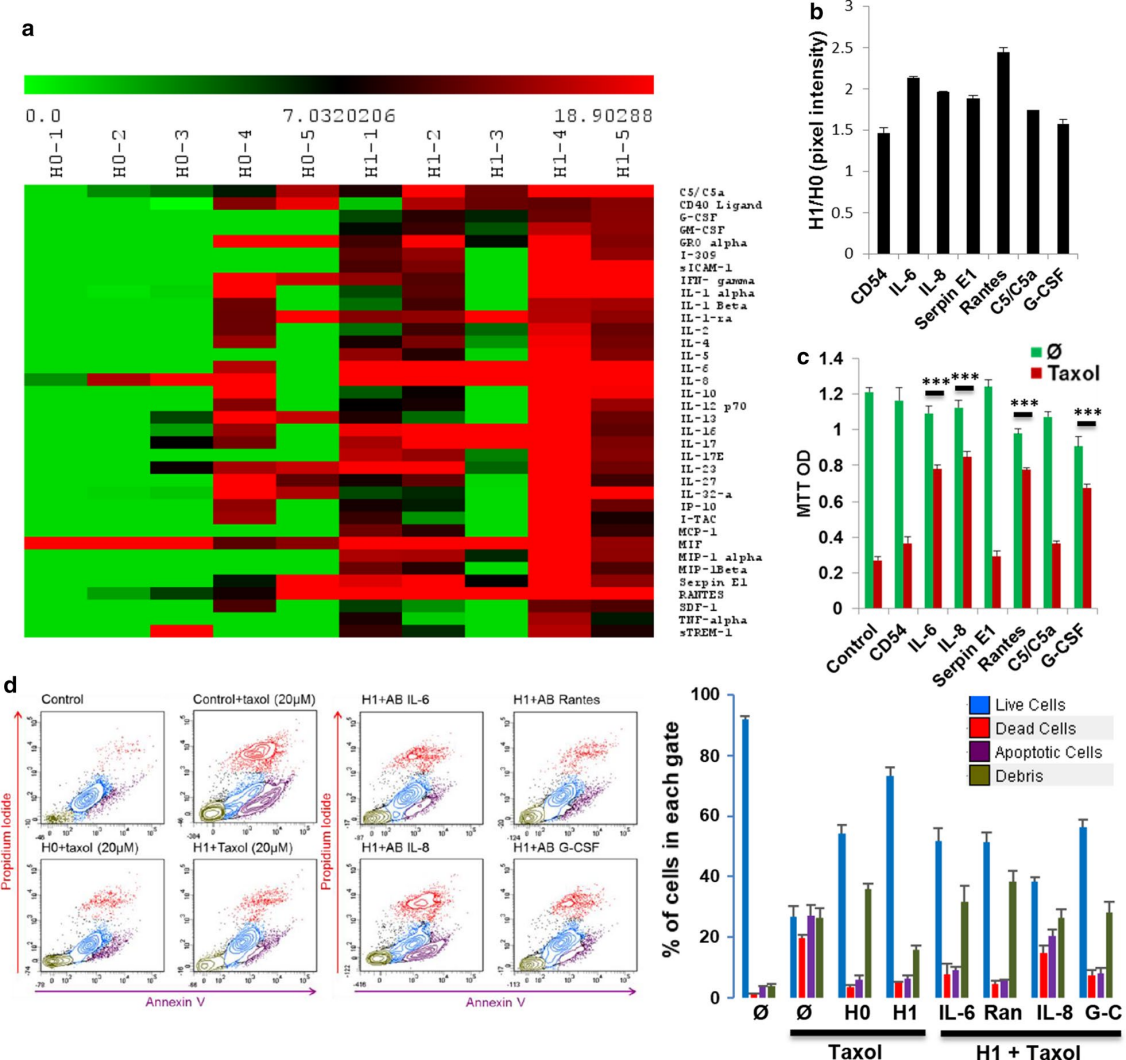
**a** Cytokine array. Cytokine arrays were performed on H0 and H1 of five patients (numbered 1–5 on the figure). Each column in the cluster graphics represents one sample and each line a cytokine. The intensity ranges from green (less expressed) to red (more expressed). **b** Pixel density of selected cytokines. The pixel density of the most expressed cytokines in H1 are represented on the histogram normalized with the H0 pixel density. **c** MTT assay. APOCC were pre-treated for 24 h with human recombinant cytokines (CD54, IL-6, IL-8, Serpin E1, Rantes, C5/C5a or G-CSF) prior to Taxol treatment (20 µM). After 48 h a MTT assay was performed. The histogram represents the mean OD MTT. **d** Cell viability. APOCC were pre-incubated with H0 or H1 prior Taxol (20 µM) treatment (left panel). APOCC pre-incubated with H0 were also incubated with specific blocking antibodies for IL-6, IL-8, Rantes or G-CSF prior Taxol (20 µM) treatment (right panel). The percentage of live cells (blue), dead cells (red), apoptotic cells (purple) and debris (green) are represented in the histogram on the right

To investigate factors responsible for the phenotypic effect of H1, we performed a cytokine array on H0 and H1 media from five different patients (Fig. 2a). We noticed differences in peritoneal basal inflammation status at H0 between patients. Patients 4 and 5 displayed a higher inflammatory profile at the beginning of the surgery (PH0) resulting in higher level of the cytokines at H1. This discordance could not be explained by demographic or pre-operative parameters. Among the 36 cytokines tested, many cytokines involved in the inflammatory response (Cd54, IL-6, IL-8, serpin E1 and Rantes), cell proliferation and negative regulation of cell death (G-CSF and C5/C5a) were significantly up regulated in H1 compared to H0 (p < 0.05, Fig. 2b). We then pre-treated APOCC for 24 h with human recombinant cytokines prior to Taxol therapy (20 µM) and demonstrated increased survival with IL-6, IL-8, Rantes and G-CSF (Fig. 2c). To confirm the role these different cytokines in H1 mediated resistance, we pre-incubated APOCC in H0 and H1 with specific blocking antibodies for 24 h (Fig. 2d left panel). As illustrated previously H0 and H1 increased survival of APOCC compared to controls (26.76% ± 2.6 of live cells in the control, 54.41% ± 2.2 with H0 and 73.31% ± 1.2 with H1). bAB against IL-6, IL-8, Rantes or G-CSF significantly reduced chemoresistance induced by H1 (live cells ranging from 56.39% ± 2.5 with G-CSF bAB to 38.48% ± 1.3 with IL-8 bAB; Fig. 2d right panel). Among all cytokines tested IL-8 bAB induced an apoptosis rate comparable to control conditions (27.18% ± 3.5 of apoptotic cells in the control and 20.33% ± 2.3 with IL-8 bAB).

### IL-8 in H1 induces resistance to apoptosis

**Fig. 3:**
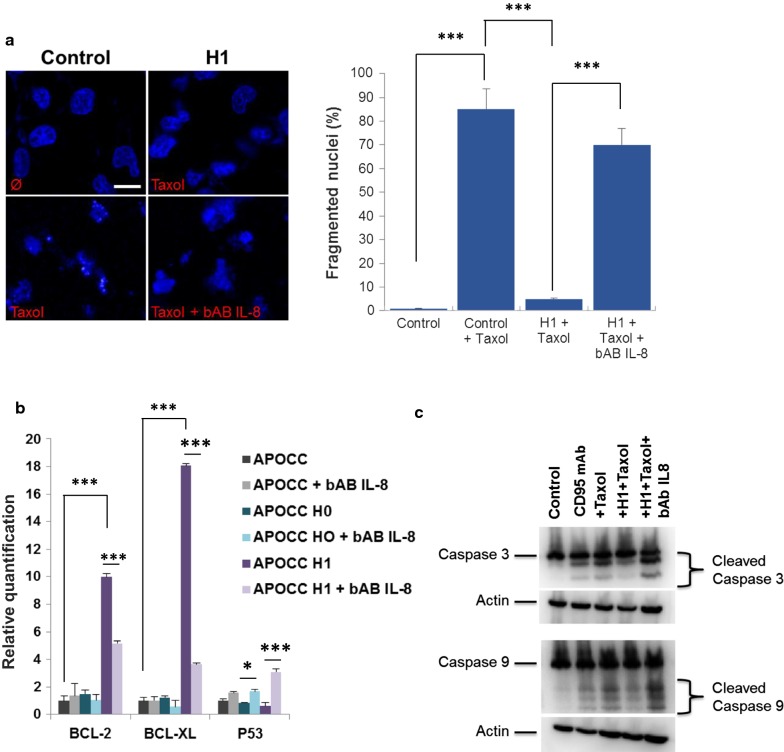
**a** Nucleus fragmentation. APOCC alone (control), pre-incubated with H1 or pre-incubated with H1+ a blocking antibody for IL-8 (bAB IL-8) were treated with Taxol (20 µM). Confocal microscopy was performed on the cells stained with DAPI. Pictures are representative of the nucleus in the well. Scale bar: 5 µm. **b** The relative quantification of apoptosis genes was performed by real-time qPCR on APOCC after different treatment (H0, H1, bAB IL-8). Relative transcript levels are represented as the log10 of ratios between the two subpopulations of their 2^−ΔΔCp^ real-time PCR values. **c** APOCC cells pre-incubated or not with H1 with or without bAB IL-8 for 24 h were treated with Taxol (20 µM). Caspase and cleaved Caspase 3 and 9 were assessed by western blot. Induced apoptosis on APOCC using an anti-Fas receptor (CD95) monoclonal antibody (mAb) was used as a positive control

Cells incubated with H1 and treated by taxol displayed less DNA fragmentation as shown by DAPI staining (85% are fragmented in the control compare to 5% only when treated with PH1; Fig. 3a). When cells incubated with H1 were treated with IL8-bAB Taxol induced DNA fragmentation was restored (70%). We examined the expression levels of anti-apoptosic proteins Bcl2, Bcl-XL and p53 in APOCC incubated with H0 or H1 and treated with IL-8-bAB (Fig. 3b). While Bcl2 and Bcl-XL were both up regulated in APOCC exposed to H1 compared to H0, IL-8-bAB inhibited the increased expression of these anti-apoptotic proteins. Concordantly H1 treatment decreased p53 expression compared to H0 and controls (40% decrease compared to 15%, respectively; p < 0.01). P53 plays a pro-apoptotic role in Taxol-induced cell death. Therefore p53 decreased expression with H1 treatment seems to be concordant with induced resistance to apoptosis [23]. This effect was inhibited by IL-8-bAB treatment.

To investigate the downstream effectors of P53 and bcl-2 family we investigated the activation of caspases under Taxol and H1 treatment (Fig. 3c). APOCC cells pre-incubated with H1 for 24 h were treated by Taxol or anti-Fas receptor (CD95) monoclonal antibody (mAb) as positive control for apoptosis. Western blot for caspase 3 and 9 showed cleaved caspase 3 and 9 in positive controls as well as cells treated with Taxol. Pre-incubation with H1 prevented caspase 3 and 9 cleavage. The effect of H1 was inhibited by IL-8-bAB.

### H1 IL-8 induces resistance to apoptosis through AKT

**Fig. 4:**
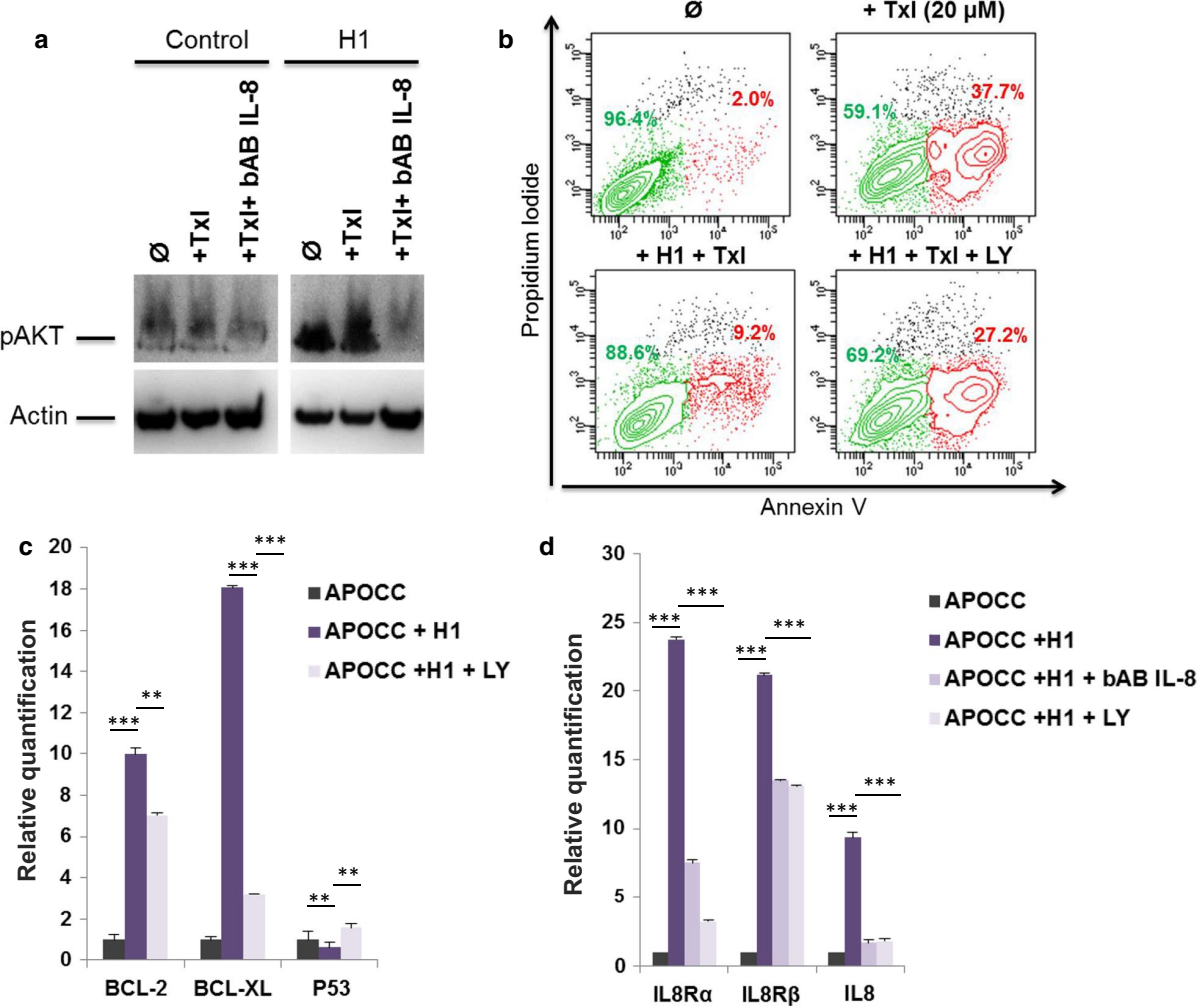
**a** Phospho-AKT western blot. APOCC cells pre-incubated or not with H1 with or without bAB IL-8 for 24 h were treated with Taxol (20 µM). Phospho-AKT was assessed by western blot. **b** Apoptosis assay. APOCC cells pre-incubated or not with H1 with or without an Akt inhibitor (LY294002) for 24 h were treated with Taxol (20 µM). Apoptosis was evaluated by flow cytometry using an apoptosis array. Live cells are represented in green and apoptotic cells in red. **c** The relative quantification of apoptosis genes was performed by real-time qPCR on APOCC before or after treatment with H1 or H1 + LY294002. Relative transcript levels are represented as the log10 of ratios between the two subpopulations of their 2^−ΔΔCp^ real-time PCR values. **d** The relative quantification of IL-8 and IL-8 receptor genes was performed by real-time qPCR on APOCC before or after treatment with H1, H1 bAB IL-8 or H1 + LY294002. Relative transcript levels are represented as the log10 of ratios between the two subpopulations of their 2^−ΔΔCp^ real-time PCR values

IL8 effect is mediated by different pathways including the Pi3-K/akt pathway. H1 incubated OCCs displayed an AKT phosphorylation which could be blocked using concomitant IL-8-bAB treatment (Fig. 4a). When we inhibited the AKT pathway (LY294002) we showed a decrease in H1 induced resistance to apoptosis (Fig. 4b). Concordantly Akt inhibition reversed the effect of H1 on Bcl2, Bcl-XL and p53 expressions (Fig. 4c).

As illustrated previously in other models we wondered if H1 was able to induce an IL-8 autocrine loop in OCC that could participate to a pro-tumoral niche. We showed an increase expression of IL-8 receptor α and β as well as IL-8 secretion in APOCC, treated with H1 suggesting the induction of a tumor autonomous IL8 autocrine loop by H1 cytokines. IL-8 blockade or AKT inhibition using LY294002 resulted in the inhibition of this autocrine loop (Fig. 4d).

### IL8 overexpression in ovarian cancer is associated with chemioresistance in vivo and impacts survival

We reviewed tumor samples from 32 patients with advanced ovarian cancer referred to Tenon Hospital from January 2004 to July 2011. They all received platinium and taxane based neoadjuvant chemotherapy. The mean overall survival (OS) and disease free survival (DFS) of our study population were 54.8 and 22 months, respectively.

Sixteen patients displayed chemoresistance as defined by a recurrence within 6 months after initial treatment. No difference was observed between chemoresistant and chemosensitive patients regarding demographic and clinical parameters. Tumoral expression of IL8 was significantly higher in chemoresistant women (31% versus 11%, respectively; p = 0.003, Table 1). On multivariate analysis, IL8 expression was the only independent risk factor for chemoresistance (HR = 1.1, p = 0.016). We determined an optimal cut-off of 40% (p = 0.005) for IL8 staining in tumor samples retrieved during debulking surgery, using iterative log-rank test to maximize its prognostic value.

Patients were thus stratified according to IL8 tumor expression: women who displayed IL8 tumoral expression greater than 40% constituted the “high IL8” subgroup (n = 8). Remaining patients formed the “low IL8” subgroup (n = 24). We did not observe any significant difference between the two subgroups regarding clinical or treatment parameters, apart from CA125 value at baseline that was significantly higher in the “high IL8” subgroup (Table 2). “High IL8” patients displayed poorer survival outcomes compared to “low IL8” women. OS was 32.4 versus 63.3 months, respectively (p = 0.009) (Table 2). DFS was 12.1 versus 35.6 months, respectively (p = 0.001). These findings underline the prognostic significance of IL8 tumoral expression, and its association with chemoresistance in patients. Interestingly high IL8 expression was associated to earlier recurrence after complete treatment suggesting its role not only in primary resistance but also to the persistence of a residual disease responsible for the recurrences.

**Table 2:** Comparative demographics according to tumoral expression of IL8

	Study population (n = 32)	High IL8 group (n = 8)	Low IL8 group (n = 24)	p-value
Age (years)	62.4 (± 10.1)	61.4 (± 12.6)	62.8 (± 9.4)	0.75
BMI	23.7 (± 3.9)	24.4 (± 4.5)	23.7 (± 3.9)	0.68
CA 125 (U/ml) at baseline	1878.5 (± 2402.6)	3615.6 (± 4010.7)	1299.6 (± 1218.2)	0.01*
Histological type				
Serous	81.3% (n = 26)	87.5% (n = 7)	79.2% (n = 19)	0.67
Mucinous	3.1% (n = 1)	0	4.2% (n = 1)	
Endometrioid	6.2% (n = 2)	0	8.3% (n = 2)	
Undifferentiated	9.4% (n = 3)	12.5% (n = 1)	8.3% (n = 2)	
FIGO stage				
IIIC	87.5% (n = 28)	75.0% (n = 6)	91.7% (n = 22)	0.08
IV	12.5% (n = 4)	25.0% (n = 2)	8.3% (n = 2)	
Number of NAC courses	4 (3–7)	4 (3–6)	4 (3–7)	0.32
Total number of chemotherapy courses	6 (3–12)	6 (3–10)	6 (3–12)	0.24
Delay between diagnosis and surgery (months)	3.7 (2.6–9.3)	3.8 (2.9–7.0)	3.7 (2.6–9.3)	0.42
Completeness of cytoreduction score^a^				
CC-0	78.1% (n = 25)	62.5% (n = 5)	83.3% (n = 20)	0.22
CC-1	3.1% (n = 1)	0	4.2% (n = 1)	
CC-2	12.5% (n = 4)	25.0% (n = 2)	8.3% (n = 2)	
CC-3	6.3% (n = 2)	12.5% (n = 1)	4.2% (n = 1)	
Duration of surgical procedure (min)	389 (± 127)	360 (± 132)	401 (± 127)	0.52
Disease free survival (months)	29.4 (± 5.1)	12.2 (± 1.0)	35.6 (± 6.5)	0.001**
Overall survival (months)	54.8 (± 6.1)	32.4 (± 7.3)	63.3 (± 7.1)	0.009**
Chemoresistance				
Yes	57.2% (n = 16)	100.0% (n = 8)	40.0% (n = 8)	0.004**
No	42.8% (n = 12)	0	60.0% (n = 12)	

*BMI* body mass index, *NAC* neoadjuvant chemotherapy

^a^A CC-0 score indicates a complete disease removal; a CC-1 score indicates that tumor nodules persisting after cytoreduction were < 2.5 mm in diameter; a CC-2 score indicates residual tumor nodules between 2.5 mm and 25 mm in diameter; a CC-3 score indicates residual tumor nodules > 25 mm in diameter or a confluence of unresectable tumor nodules at any site within the abdomen or pelvis

## Discussion

Our results support that peritoneal response to surgical stress favors chemoresistance in ovarian cancer cells through the establishment of an autocrine IL8 loop (OCC). In our model, increased peritoneal production of IL8 is associated with resistance to apoptosis through both AKT pathway activation and OCC overexpression of IL8. In the clinical setting, we observed a detrimental impact of tumoral overexpression of IL8 on chemosensitivity and survival outcomes with a significant association to earlier recurrence, supporting the concept of a peritoneal residual niche.

To date, debulking surgery is the cornerstone in first line treatment of advanced ovarian cancer (AOC), aiming to reach complete cytoreduction. It has been demonstrated that surgical stress favors tumor growth and metastasis in AOC as in other tumor models [24–26]. Indeed, Lee et al. [16] have observed in a mouse model of ovarian cancer that surgical stress induced by laparotomy enhanced tumor growth and angiogenesis through β-adrenergic receptor signaling. They also observed increased serum concentrations of several inflammatory cytokines during the pre-operative period, but their panel did not include IL8. Other studies have evaluated the impact of surgical route on ovarian cancer growth [15, 16, 27–29]. Canis et al. [27] have provided a macroscopic evaluation of tumor growth according to the type of surgical approach (laparotomy versus laparoscopy) in a rat model of ovarian cancer. To mimic intraoperative rupture of ovarian tumor, OCC were injected at the time of surgery. They observed that the mean dissemination score 2 weeks after surgery was higher in the laparotomy group. Most implants were found along the midline abdominal scare. They also assessed the impact of surgical peritoneal environment in a pre-implanted ovarian cancer mice model [28, 29]. Mice were stratified according to surgical route: laparotomy, laparoscopy and anesthesia alone. Macroscopic evaluation revealed a significant increase in tumor load in the laparotomy group within 1st week following surgery. However, on postoperative day 14, no difference was observed between groups regarding the dissemination score. In contrast, pathological examination demonstrated an increased incidence of muscle layers invasion in the laparotomy group. While molecular analysis showed higher levels of uPAR and cMet mRNA in tumor implants within 1st week following surgical stress, no difference was observed on day 14, suggesting a transient impact of the surgical route in cancer progression. These results suggest that severity of surgical trauma is correlated with tumor load. However, laparotomy remains the standard surgical approach in patients with extended carcinomatosis since laparoscopy may underestimate disease extent [30]. Usually adjuvant chemotherapy is initiated a month to 6 weeks after the debulking surgery. We know that patients with CCO surgery still harbor a microscopic disease, and the absence of treatment during a month constitute a window for cancer cells to define a sanctuary and eventually resist to chemotherapy. The recent trial demonstrating a survival advantage for HIPEC in the setting of interval surgery could illustrate the impact of peritoneal treatment in ovarian cancer surgery [31].

In our settings, surgical stress promoted OCC resistance to taxane based chemotherapy through peritoneal secretion of IL8. IL8 is a multifunctional chemokine, secreted by various cell types, including monocytes, neutrophils and endothelial, mesothelial and tumor cells [32]. It has been demonstrated that autocrine production of IL8 by OCC was associated with increased growth, adhesion, invasion, angiogenic potential and resistance to platinium and taxane based chemotherapy [17, 32, 33]. In our model, paracrine secretion of IL8 by stressed peritoneum might contribute to a shift in OCC phenotype toward a resistant profile. This study constitutes a preliminary step toward a more comprehensive study of per and post-operative peritoneal physiology. Indeed, for simplifications and reproducibility, we have evaluated patients with no peritoneal carcinosis and at a single time-point during surgery below the normal duration of an ovarian cancer debulking surgery. We can also hypothesize that the inflammatory response of the peritoneum to the surgical stress will be variable between patients and at different time-points during surgery. The comprehensive analysis of multiple peritoneal biopsy will allow us to acquire specific knowledge of the pre and post-operative peritoneal environment and set-up specific pre-operative procedures (humidification of the peritoneum, better control of the temperature) to inhibit the establishment of a pro-tumoral niche.

## Conclusion

In this era of precision medicine, we should consider a new paradigm with a global vision of the surgical effort. While extensive pre-operative work-up is performed to select patients, no cancer specific measures/process are performed in the immediate post-operative period when the post-operative cytokine contexture might play a role in setting-up a niche for tumor cells and subsequently affecting prognosis. Identifying the factors responsible of such phenomenon and targeting them in the context of the surgical procedure might lead to the development of precision surgery where targeted treatment will be used in combination with debulking procedure to obtain better tumor control.

## Additional file

**Additional file 1: Figure S1.** **A**. Phase contrast microscopy imaging. Ovarian cancer cells (SKOV3and OVCAR3) treated with Taxol (20 µM) for 24 h in presence of H0 (middle picture), H1 (right picture) or nothing (left picture). Scale bar: 500 µm. **B**. MTT assay. SKOV3 (green) and OVCAR3 (red) were treated with Taxol (20 µM) in presence of H0 or H1. After 48 h a MTT assay was performed. The histogram represents the mean OD MTT. **C**. Proliferation assay. OVCAR3 and SKOV3 were plated and counted every 2 days in presence or not of H0 or H1 during 6 days.

## References

[1] Rizzuto I, Stavraka C, Chatterjee J, Borley J, Hopkins TG, Gabra H, Ghaem-Maghami S, Huson L, Blagden SP (2015). Risk of ovarian cancer relapse score: a prognostic algorithm to predict relapse following treatment for advanced ovarian cancer. Int J Gynecol Cancer.

[2] Luyckx M, Leblanc E, Filleron T, Morice P, Darai E, Classe JM, Ferron G, Stoeckle E, Pomel C, Vinet B (2012). Maximal cytoreduction in patients with FIGO stage IIIC to stage IV ovarian, fallopian, and peritoneal cancer in day-to-day practice: a retrospective French multicentric study. Int J Gynecol Cancer.

[3] Vidal F, Guerby P, Luyckx M, Haddad P, Stoeckle E, Morice P, Leblanc E, Lecuru F, Darai E, Classe JM (2016). Are early relapses in advanced-stage ovarian cancer doomed to a poor prognosis?. PLoS ONE.

[4] Sugarbaker PH (2001). Review of a personal experience in the management of carcinomatosis and sarcomatosis. Jpn J Clin Oncol.

[5] Touboul C, Lis R, Al Farsi H, Raynaud CM, Warfa M, Althawadi H, Mery E, Mirshahi M, Rafii A (2013). Mesenchymal stem cells enhance ovarian cancer cell infiltration through IL6 secretion in an amniochorionic membrane based 3D model. J Transl Med.

[6] Touboul C, Vidal F, Pasquier J, Lis R, Rafii A (2014). Role of mesenchymal cells in the natural history of ovarian cancer: a review. J Transl Med.

[7] Pasquier J, Abu-Kaoud N, Abdesselem H, Madani A, Hoarau-Vechot J, Thawadi HA, Vidal F, Couderc B, Favre G, Rafii A (2015). SDF-1alpha concentration dependent modulation of RhoA and Rac1 modifies breast cancer and stromal cells interaction. BMC Cancer.

[8] Pasquier J, Guerrouahen BS, Al Thawadi H, Ghiabi P, Maleki M, Abu-Kaoud N, Jacob A, Mirshahi M, Galas L, Rafii S (2013). Preferential transfer of mitochondria from endothelial to cancer cells through tunneling nanotubes modulates chemoresistance. J Transl Med.

[9] Lis R, Capdet J, Mirshahi P, Lacroix-Triki M, Dagonnet F, Klein C, Mirshahi M, Fournie JJ, Rafii A, Poupot M (2010). Oncologic trogocytosis with Hospicells induces the expression of N-cadherin by breast cancer cells. Int J Oncol.

[10] Rafii A, Mirshahi P, Poupot M, Faussat AM, Simon A, Ducros E, Mery E, Couderc B, Lis R, Capdet J (2008). Oncologic trogocytosis of an original stromal cells induces chemoresistance of ovarian tumours. PLoS ONE.

[11] Pasquier J, Gosset M, Geyl C, Hoarau-Vechot J, Chevrot A, Pocard M, Mirshahi M, Lis R, Rafii A, Touboul C (2018). CCL2/CCL5 secreted by the stroma induce IL-6/PYK2 dependent chemoresistance in ovarian cancer. Mol Cancer.

[12] Matsuzaki S, Botchorishvili R, Jardon K, Maleysson E, Canis M, Mage G (2011). Impact of intraperitoneal pressure and duration of surgery on levels of tissue plasminogen activator and plasminogen activator inhibitor-1 mRNA in peritoneal tissues during laparoscopic surgery. Hum Reprod.

[13] Canis M, Matsuzaki S, Bourdel N, Jardon K, Cotte B, Botchorishvili R, Rabischong B, Mage G (2007). Peritoneum and laparoscopic environment. Bull Cancer.

[14] Zhu P, Miao W, Gu F, Xing C (2018). Changes of serum and peritoneal inflammatory mediators in laparoscopic radical resection for right colon carcinoma. J Minim Access Surg.

[15] Lee JW, Park YA, Cho YJ, Kang KH, Choi JJ, Lee YY, Kim TJ, Choi CH, Kim BG, Bae DS (2013). The effect of surgical wound on ovarian carcinoma growth in an animal model. Anticancer Res.

[16] Lee JW, Shahzad MM, Lin YG, Armaiz-Pena G, Mangala LS, Han HD, Kim HS, Nam EJ, Jennings NB, Halder J (2009). Surgical stress promotes tumor growth in ovarian carcinoma. Clin Cancer Res.

[17] Wang Y, Qu Y, Niu XL, Sun WJ, Zhang XL, Li LZ (2011). Autocrine production of interleukin-8 confers cisplatin and paclitaxel resistance in ovarian cancer cells. Cytokine.

[18] Ghiabi P, Jiang J, Pasquier J, Maleki M, Abu-Kaoud N, Rafii S, Rafii A (2014). Endothelial cells provide a notch-dependent pro-tumoral niche for enhancing breast cancer survival, stemness and pro-metastatic properties. PLoS ONE.

[19] Ghiabi P, Jiang J, Pasquier J, Maleki M, Abu-Kaoud N, Halabi N, Guerrouahen BS, Rafii S, Rafii A (2015). Breast cancer cells promote a notch-dependent mesenchymal phenotype in endothelial cells participating to a pro-tumoral niche. J Transl Med.

[20] Al Thawadi H, Abu-Kaoud N, Al Farsi H, Hoarau-Vechot J, Rafii S, Rafii A, Pasquier J (2016). VE-cadherin cleavage by ovarian cancer microparticles induces beta-catenin phosphorylation in endothelial cells. Oncotarget.

[21] Pasquier J, Rioult D, Abu-Kaoud N, Marie S, Rafii A, Guerrouahen BS, Le Foll F (2013). P-glycoprotein-activity measurements in multidrug resistant cell lines: single-cell versus single-well population fluorescence methods. Biomed Res Int.

[22] Bonneau C, Rouzier R, Geyl C, Cortez A, Castela M, Lis R, Darai E, Touboul C (2015). Predictive markers of chemoresistance in advanced stages epithelial ovarian carcinoma. Gynecol Oncol.

[23] Kim JH, Yoon EK, Chung HJ, Park SY, Hong KM, Lee CH, Lee YS, Choi K, Yang Y, Kim K (2013). p53 acetylation enhances Taxol-induced apoptosis in human cancer cells. Apoptosis.

[24] Kim R (2018). Effects of surgery and anesthetic choice on immunosuppression and cancer recurrence. J Transl Med.

[25] Behrenbruch C, Shembrey C, Paquet-Fifield S, Molck C, Cho HJ, Michael M, Thomson BNJ, Heriot AG, Hollande F (2018). Surgical stress response and promotion of metastasis in colorectal cancer: a complex and heterogeneous process. Clin Exp Metastasis.

[26] Goldstein MR, Mascitelli L (2011). Surgery and cancer promotion: are we trading beauty for cancer?. QJM.

[27] Canis M, Botchorishvili R, Wattiez A, Mage G, Pouly JL, Bruhat MA (1998). Tumor growth and dissemination after laparotomy and CO2 pneumoperitoneum: a rat ovarian cancer model. Obstet Gynecol.

[28] Matsuzaki S, Azuar AS, Mage G, Canis M (2010). Impact of the surgical peritoneal environment on pre-implanted tumors on a molecular level: a syngeneic mouse model. J Surg Res.

[29] Azuar AS, Matsuzaki S, Darcha C, Dechelotte PJ, Pouly JL, Mage G, Canis M (2009). Impact of surgical peritoneal environment on postoperative tumor growth and dissemination in a preimplanted tumor model. Surg Endosc.

[30] Le Brun JF, Ferron G, Vaysse C, Baujat M, Leguevaque P, Filleron T, Querleu D (2012). Laparoscopic observation of the diaphragm undersurface in the staging of peritoneal carcinomatosis: comparison of three optical systems. Eur J Obstet Gynecol Reprod Biol.

[31] van Driel WJ, Koole SN, Sikorska K, Schagen van Leeuwen JH, Schreuder HWR, Hermans RHM, de Hingh I, van der Velden J, Arts HJ, Massuger L (2018). Hyperthermic intraperitoneal chemotherapy in ovarian cancer. N Engl J Med.

[32] Wang Y, Xu RC, Zhang XL, Niu XL, Qu Y, Li LZ, Meng XY (2012). Interleukin-8 secretion by ovarian cancer cells increases anchorage-independent growth, proliferation, angiogenic potential, adhesion and invasion. Cytokine.

[33] Stronach EA, Cunnea P, Turner C, Guney T, Aiyappa R, Jeyapalan S, de Sousa CH, Browne A, Magdy N, Studd JB (2015). The role of interleukin-8 (IL-8) and IL-8 receptors in platinum response in high grade serous ovarian carcinoma. Oncotarget.

