# Supplementary figures and images for: Surgical peritoneal stress creates a pro-metastatic niche promoting resistance to apoptosis via IL-8
Source: J Transl Med. 2018 Oct 3;16:271. doi: 10.1186/s12967-018-1643-z (PMC6171219; doi:10.1186/s12967-018-1643-z)

Figure S1

A

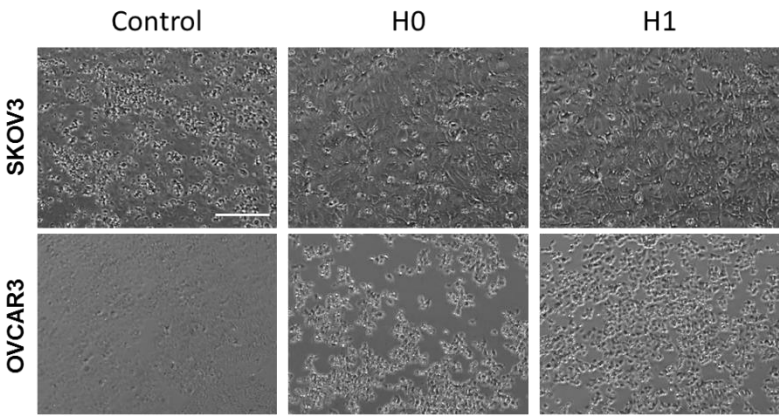

B

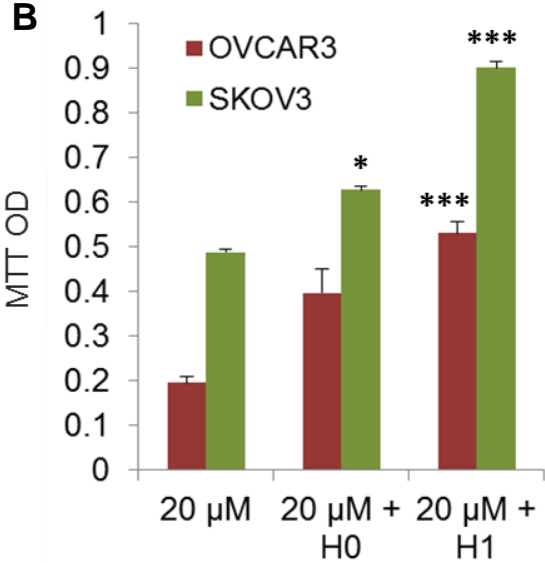

C

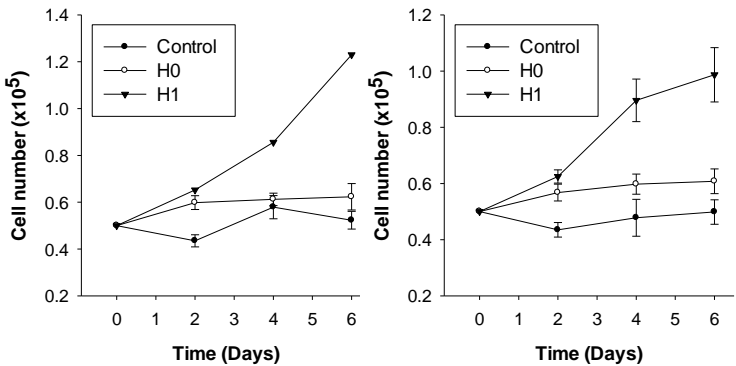

Supplement: Supplementary file 1 — Additional file 1: Figure S1. A. Phase contrast microscopy imaging. Ovarian cancer cells (SKOV3and OVCAR3) treated with Taxol (20 µM) for 24 h in presence of H0 (middle picture), H1 (right picture) or nothing (left picture). Scale bar: 500 µm. B. MTT assay. SKOV3 (green) and OVCAR3 (red) were treated with Taxol (20 µM) in presence of H0 or H1. After 48 h a MTT assay was performed. The histogram represents the mean OD MTT. C. Proliferation assay. OVCAR3 and SKOV3 were plated and counted every 2 days in presence or not of H0 or H1 during 6 days. [file 12967_2018_1643_MOESM1_ESM.pdf]
